# LncRNA SNHG14 promotes cell proliferation and invasion in colorectal cancer through modulating miR-519b-3p/DDX5 axis

**DOI:** 10.7150/jca.55495

**Published:** 2021-06-11

**Authors:** Xiaoyuan Wang, Peng Yang, Dongsheng Zhang, Ming Lu, Chi Zhang, Yueming Sun

**Affiliations:** 1 Department of General Surgery, The Second Affiliated Hospital Of Nanjing Medical University, Nanjing, Jiangsu Province, China.; 2 Department of General Surgery, The First Affiliated Hospital Of Nanjing Medical University, Nanjing, Jiangsu Province, China.

**Keywords:** lncRNA SNHG14, miR-519b-3p, DDX5, colorectal cancer

## Abstract

Numbers of studies suggest that long non-coding RNAs (lncRNAs) exert an important role in cancer progression. It is reported that lncRNA SNHG14 (SNHG14) promotes cell proliferation and invasion in many cancers. However, the underlying molecular mechanism of SNHG14 in colorectal cancer (CRC) remains unclear. In our study, we found that SNHG14 is highly expressed in CRC tissues and cells, especially in SW480 and HT-29 cells. In addition, sh-SNHG14 inhibits cell proliferation, cell migration and invasion, promotes cell apoptosis in CRC cell lines. Furthermore, we found that SNHG14 functions as a sponge for miR-519b-3p, while the DEAD box protein 5 (DDX5) is a downstream target gene of miR-519b-3p, and the functions of miR-519b-3p inhibitors on the CRC progression could be rescued by downregulation of DDX5. Our findings suggest that SNHG14 promotes the CRC progression by miR-519b-3p/DDX5 axis, implying the promising therapeutic target of SNHG4 for CRC patients.

## Introduction

Colorectal cancer (CRC) causes nearly 700,000 deaths each year, making it the world's fourth killer cancer (after lung, liver and stomach) 1. In recent years, the treatment methods of CRC have made great progress, but the mortality rate of CRC patients is still high. Therefore, it has become a research hotspot to explore the potential molecular mechanism of CRC occurrence and development, and search for new molecular therapeutic targets for CRC.

Long non-coding RNAs (lncRNAs) are a type of RNAs whose transcripts are more than 200 nucleotides in length, and have no/little potentials in coding proteins 2, 3. It is reported that lncRNAs play extensive regulatory roles in life activities and the occurrence and development of diseases (especially cancer, neurological diseases) 4. Numbers of studies demonstrated that lncRNAs exert important roles in the regulation of cell proliferation, migration, invasion and apoptosis 5-7. In recent years, studies have found lncRNAs act as important regulators in the development of CRC. For instance, lncRNA MALAT1 overexpression promotes endothelial cell formation and HIF-1 α protein expression, and participates in the occurrence and development of colorectal cancer by promoting the colorectal cancer cells mediated angiogenesis 8. LncRNA CCAL regulates CRC occurrence and development via activating Wnt/β-catenin signaling pathway through inhibition of activator protein 2α 9. Additionally, the modulation of lncRNA FLANC expression affects the cell growth, apoptosis, migration, angiogenesis and metastasis formation ability of CRC cells 10.

Recent studies indicated that lncRNA SNHG14 promotes hepatocellular carcinoma progression by regulating miR-4673/SOCS1 11. LncRNA SNHG14 silencing inhibited non-small cell lung cancer progression via miR-34a/HMGB1 axis and promoted NSCLC cell cisplatin sensitivity 12. Nevertheless, the function and molecular mechanism of lncRNA SNHG14 in CRC remains unclear.

In our study, we aimed to explore the function and potential underlying mechanism of SNHG14 in CRC. Subsequently, the influences of SNHG14 knockdown on CRC progression. Finally, we found that SNHG14 promotes cell proliferation and invasion in colorectal cancer through modulating miR-519b-3p/DDX5 axis. Our study will provide an experimental basis for the molecular mechanism of CRC and offer therapeutic targets against the CRC.

## Materials and methods

### Clinical samples

A total of 30 paired CRC tumor tissues and adjacent non-tumor tissues were obtained from the Second Affiliated Hospital of Nanjing Medical University for this study. Patients older than 18 years and those who had undergone R0 resection were eligible for the study; however, those with metastasis, a concurrent diagnosis of familial adenomatous polyposis, lynch syndrome or irritable bowel disease (IBD), or metachronous or synchronous CRC were excluded from the analysis. All patients did not get treatment. Each patient had signed a written informed consent. The First School of Clinical Medicine, The First Affiliated Hospital of Nanjing Medical University Ethics Committees approved this work. The Ethics Committees approved protocol number was 2019-SRFA-131. In addition, detailed clinic parameters had been shown in the Table 1.

### Establishment of colorectal cancer model

20 male NOD-SCID mice aged at 8 weeks were used to establish model in the experiment. These mice were purchased from Charles River Laboratories (Beijing, China). The transfected HT-29 cells (5 × 10^6^ cells in 100μL PBS) were injected subcutaneously into the forelimbs of nude mice. All the mice were observed every 2 days for at least 7 weeks. All procedures were conformed to the Guide for the Care and Use of Laboratory Animal published by the US National Institutes of Health (NIH publication, 8th edition, 2011), and approved in accordance with the Committee on Animal Care and Use of ethical committee of Nanjing Medical University. Ethical number of animal experiment is IACUC-1903018. The nude mice were housed in 12-h light/dark cycle with a free access to standard chow and tap water in a temperature-controlled room.

### Cell culture and transfection

Human CRC cell lines (SW480, HT-29, HCT-8 and DLD-1) and human normal colon epithelial cells (NCM460) were purchased from the American Type Culture Collection (ATCC; Manassas, VA, USA). All the cells were cultured in RPMI 1640 medium supplemented with 10% fetal bovine serum (FBS), 100 U mL^-1^ penicillin, and 100 g/ mL streptomycin and incubated at 5% CO_2_; 37 °C.

Short hairpin RNA (shRNA) targeting SNHG14 (sh-SNHG14), sh-DDX5 and the corresponding negative controls (sh-NC) were designed and synthesized by Thermo Fisher Scientific (Shanghai, China). MiR-519b-3p mimics, miR-519b-3p inhibitor and the corresponding negative controls (miR-NC) were purchased from Thermo Fisher Scientific. These plasmids were transfected into cells by using Lipofectamine 2000 (Invitrogen, CA, USA) according to the manufacturer's instruction.

### qRT-PCR assay

Total RNAs of the samples were extracted by Trizol reagent (Takara, Otsu, Japan) following the manufacturer's protocols. Reverse transcriptase reactions were performed using HiScript® III 1st Strand cDNA Synthesis Kit (Vazyme, Nanjing, China). All qRT-PCR experiments were performed using SYBR green reagents (Vazyme, Nanjing, China) and a qRT-PCR Detection System (Analytic, jena, Germany). The quantitative measures were obtained using the 2^-ΔΔCT^ method and were normalized to either β-actin or U6 level. The primers for RT-qPCR were as follows: hsa-SNHG14: 5′-GGGTGTTTACGTAGACCAGAACC-3′ (Forward) and 5′- CTTCCAAAAGCCTTCTGCCTTAG-3′ (Reverse); hsa-miR-519b-3p: 5′-GGTCAAGTGACACCGTCG-3′ (Forward) and 5′-TGCAGCTGGGGGTCAG-3′ (Reverse); hsa-DDX5: 5′-GGCCTGATCACAGAACCATT-3′ (Forward) and 5′-ACC ACCCTTATTCCCAAACC-3′ (Reverse); hsa-U6: 5′-GTGATCACTCCCTGCCTGAG-3′ (Forward) and 5′-GGACTTCACTGGACCAGACG-3′ (Reverse); hsa-GAPDH: 5′-CCGCATCTTCTTGTGCAGTG-3′ (Forward) and 5′-CCCAATACGGCCAAATCCGT-3′ (Reverse).

### CCK-8 assay

Cell Counting Kit8 (CCK-8; Apexbio, HOU, USA) was used to detect CRC cells growth. Briefly, SW480 and HT-29 cells (5×10^3^ cells/well) were incubated in 96-well for 24, 48 or 72 h. Then, the cells were treated with certain drugs for 24 h, and 10 μL of CCK-8 solution was subsequently added to each well. Then the plate was incubated at 37 °C for 3 h. The optical density (OD) value was measured at 450 nm by ultraviolet spectrophotometer (Thermo Fisher Scientific, Inc.).

### EdU assay

Cell proliferation was assessed by EdU assay (Solarbio, Beijing, China). SW480 and HT-29 cells (2×10^4^ cells/well) were seeded on the glass coverslips in 24‐well plates and incubated in RPMI 1640 containing 10% FBS for 24h. EdU was added to each well for 2 h. After fixed, the cells were stained by 1×Apollo for 30 min in dark. Next, DAPI was used to stain nucleus for 5 min. In the end, cells were observed under fluorescence microscope (Olympus, Tokyo, Japan).

### Western blot assay

Total proteins were extracted with ice-cold RIPA lysis buffer plus PMSF. Total protein concentrations were detected with BCA assay kit (Santa Cruz, California, USA). Prepared protein samples were separated in 10% sodium dodecyl sulfate polyacrylamide gel electrophoresis (SDS-PAGE), and transferred into 0.22 μm PVDF membranes. After blocked with skim milk, these membranes were incubated with primary antibodies at 4 °C overnights, followed by incubation with the appropriate secondary antibody for 1 h at room temperature. Finally, the enhanced chemiluminescence (ECL, Pierce, Rockford, IL) visualized this membrane. The primary antibodies were anti-PCNA, anti-Ki-67, anti-Bcl-2, anti-Bax, anti-cleaved caspase-3, anti-cleaved caspase-9, anti-Cox-2, anti-MMP-2, anti-MMP-9, anti-DDX5 and anti-β-actin (Abcam, Cambridge, UK).

### Cell apoptosis assay

Cell apoptosis was tested by the Annexin V-FITC kit (Thermo Fisher Scientific, Inc.). First, SW480 and HT-29 cells were collected, resuspended in cold PBS, centrifugated at 1000 rpm in room temperature for 5-10 min. Then, cells were incubated with 5 μL AnnexinV-Alexa Fluor 647 for 15 min and then co-incubated with 5 μL PI before detection. Finally, the apoptosis rate was analyzed via flow cytometer (Beckman Coulter, CA, USA).

### Transwell chamber assay

Cell invasion and migration were detected by transwell assay. The transwell membrane was covered with (invasion) or without (migration) matrigel. Transwell lower chamber was put 600 μL RPMI-1640 medium containing 10% FBS, added 200 mL cell suspension to the upper chamber for 24 h. After incubation for 48 h, invaded or migrated cells were fixed with methanol and stained with 0.1% crystal violet. Finally, the cells were observed under a microscope (Olympus, Tokyo, Japan) and counted.

### Luciferase reporter assay

The wild-type reporter of the 3'UTR region of DDX5 (DDX5-WT), the wild-type SNHG14 reporter (SNHG14-WT), the mutant-type SNHG14 reporter (SNHG14-Mut) and the mutant-type DDX5 reporter (DDX5-Mut) were purchased from Synthgene Biotech (Nanjing, China). SNHG14-WT or SNHG14-Mut were co-transfected with miR-519b-3p mimic or miR-NC into SW480 and HT-29 cells with Lipofectamine 2000. DDX5-WT or DDX5-Mut were also co-transfected miR-519b-3p mimic or miR-NC into SW480 and HT-29 cells. After transfection for 48 h, the relative luciferase activities of cells were measured by the dual Glo Luciferase Assay System (Promega, Shanghai, China) in accordance with the manufacturer's introductions. Renilla signals were used to normalize luciferase activity.

### HE staining

These samples were collected from sh-NC and sh-SNHG14 group. The sample paraffin sections were dewaxed and hydrated; and then slices were incubated in hematoxylin solution for 15 min followed washed with PBS. Secondly, the slices were differentiated, stained by eosin solution for 15s. In the end, the slices were dehydrated, transparentized and sealed, the images were captured under an optical microscope (Olympus, Tokyo, Japan).

### Immunohistochemistry

The sliced tumors were dewaxed, hydrated and incubated with 3% H_2_O_2_ for 10 min in order to block endogenous peroxidase activity, and washed with PBS. The slices were incubated with primary antibody against Ki-67 (1:100) overnight at 4 °C, and then incubated with secondary antibodies at room temperature for 1-2 h, washed with 1×TBST and incubated in diaminobenzidine chromogen for 5 min, and the nucleus counterstained with hematoxylin. The slices were observed under microscope (Olympus, Tokyo, Japan). Ki‐67 positive staining was developed to produce brown reaction product.

### TUNEL staining

The sample paraffin sections were dewaxed and hydrated; the endogenous peroxidase was inactivated by hydrogen peroxide. Next, tunel test solution labeled sample biotin followed incubated with DAB for 5-30 min, and then the slices were incubated in hematoxylin solution for 15 min followed washed with PBS. Finally, the slices were dehydrated transparentized, sealed and observed under optical microscope (Olympus, Tokyo, Japan).

### Statistical analysis

Data was presented as the mean ± standard deviation (SD) of at least 3 independent experiments. Student's *t*-test was performed to determine the significance between two groups, or one-way ANOVA with Bonferroni post-hoc tests for multiple groups, *P< 0.05 and **P<0.01 were considered as significant.

## Results

### Clinical parameters of enrolled CRC patients in our study

The clinicopathological information of enrolled 30 CRC patients was presented in Table 1. As shown in Table 1, we found that high SNHG14 expression was strongly correlated with tumor stage, tumor size and distant metastasis. However, there were no significant differences between age, gender and lymph node metastasis.

### SNHG14 is highly expressed in CRC tissues and cells

**Figure 1:**
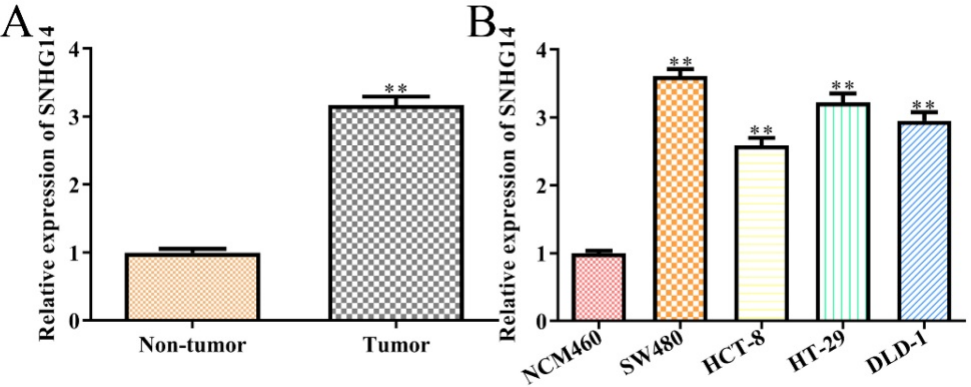
** SNHG14 is highly expressed in CRC tissues and cells. (A)** qRT-PCR showed expression of SNHG14 in tumor tissues and adjacent non-tumor tissues. **P<0.01 *vs.* Non-tumor tissue. n= 6. **(B)** qRT-PCR tested the expression of SNHG14 in CRC cell lines (SW480, HCT-8, HT-29 and DLD-1) and normal human colon epithelial cells (NCM460). Values are mean ± SEM **P<0.01 *vs.* NCM460. n=3 per group.

To detect the correlations between the expression levels of SNHG14 and the occurrence/development of CRC, we firstly analyzed the expression of SNHG14 in CRC tissues and cells, individually. We found that SNHG14 expression was significantly upregulated in CRC tissues compared with adjacent non-tumor tissues (Fig. 1A). As shown in Fig. 1B, the expression of SNHG14 was remarkably increased in CRC cells (SW480, HCT-8, HT-29 and DLD-1) compared to normal human colon epithelial cells (NCM460). Considering that the SNHG14 expression was dramatically higher in SW480 and HT-29 cells than other CRC cells (Fig. 1B). Therefore, SW480 and HT-29 cells were selected for subsequent experiments.

### Knockdown of SNHG14 inhibits the proliferation and promotes the apoptosis of CRC cells

**Figure 2:**
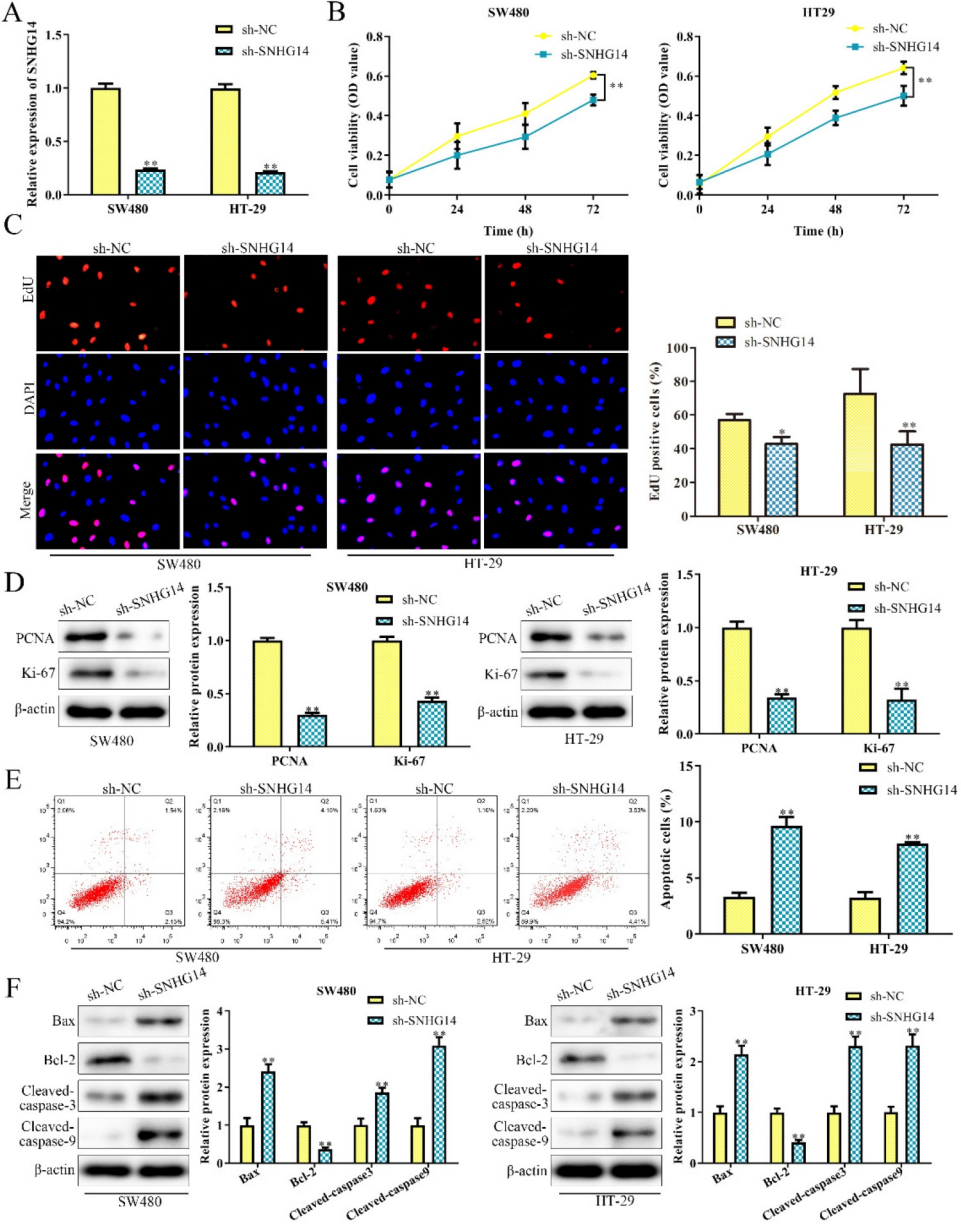
** Sh-SNHG14 inhibits cell proliferation and promotes cell apoptosis in CRC.** SW480 and HT-29 cells were transfected with sh-SNHG14. **(A)** qRT-PCR analysis to detect the knockdown efficiency of SNHG14. **(B)** CCK-8 assays for the cell proliferation at the indicated time points (24, 48 and 72h). **(C)** EdU assays for the cell proliferation. **(D)** Western blot analysis of the expression levels of proliferation-associated proteins (PCNA and Ki-67). **(E)** Flow cytometry analyzed the apoptosis rate. **(F)** Western blot analysis of the expression levels of apoptosis associated proteins (Bax, Bcl-2, Cleaved caspase-3 and Cleaved caspase-9). Values are mean ± SE, *P<0.05, **P<0.01 *vs.* sh-NC, n=3 per group.

To investigate the biological function of SNHG14 in CRC, sh-SNHG14 and its scramble control (sh-NC) were transfected into SW480 and HT-29 cells, respectively. The knockdown efficiency of sh-SNHG14 in SW480 and HT-29 cells were validated by qRT-PCR analysis and were presented in Fig. 2A. Functionally, CCK-8 and EdU assays showed that sh-SNHG14 significantly inhibited the proliferation of SW480 and HT-29 cells (Fig. 2B-C). Furthermore, PCNA and Ki-67, the proliferation-related proteins, were markedly downregulated by sh-SNHG14 in SW480 and HT-29 cells (Fig. 2D). Next, flow cytometry assay indicated that sh-SNHG14 promoted cell apoptosis in SW480 and HT-29 cells (Fig. 2E). In addition, the expression of apoptosis-related proteins was examined by western blot analysis. We found that sh-SNHG14 reduced the expression level of anti-apoptotic protein (Bcl-2), while elevated the expression levels of pro-apoptosis proteins (Bax, Cleaved caspase-3 and Cleaved caspase-9) in SW480 and HT-29 cells (Fig. 2F). These results indicated that sh-SNHG14 inhibits the proliferation and promotes the apoptosis of CRC cells.

### Knockdown of SNHG14 inhibits migration and invasion of CRC cells

**Figure 3:**
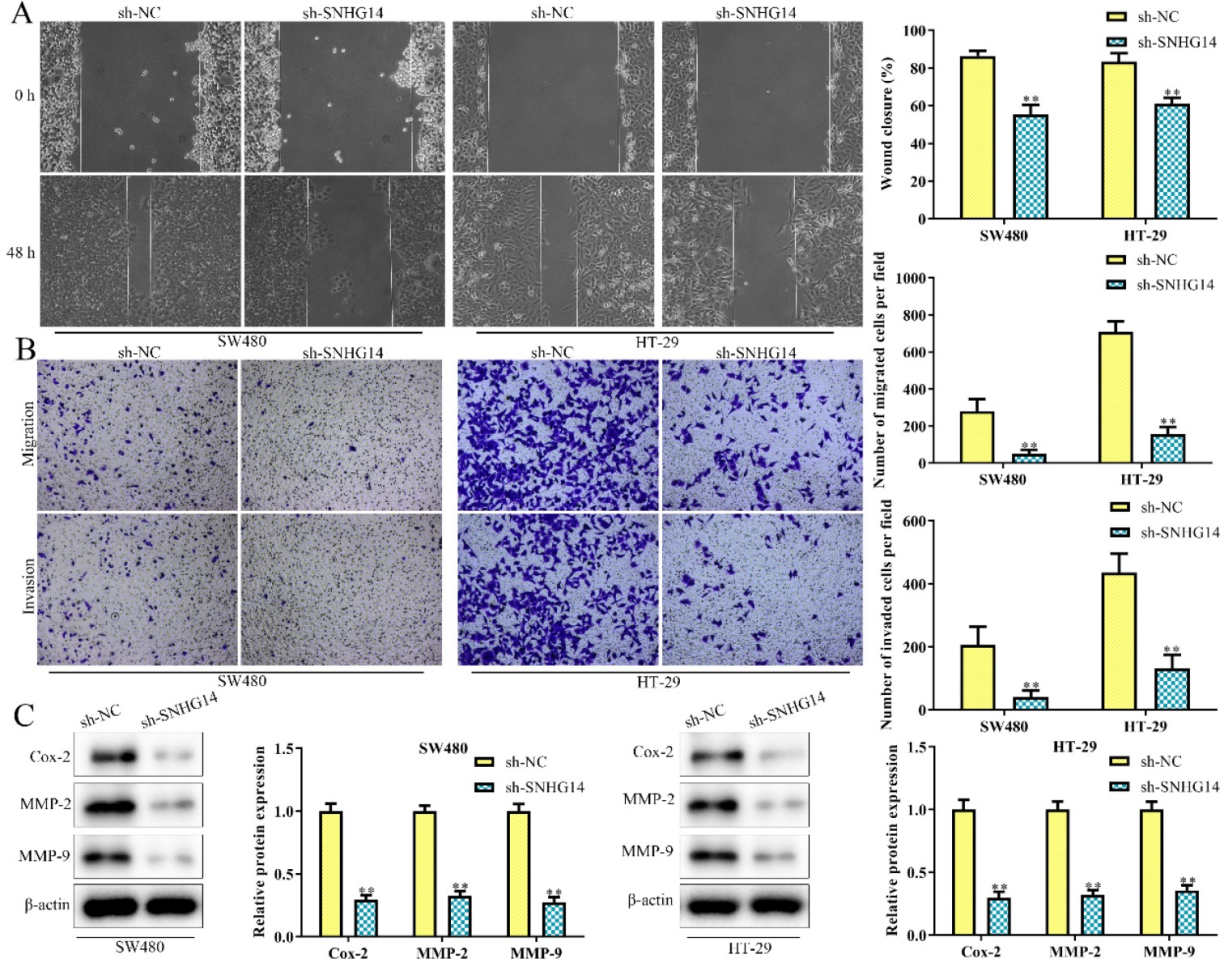
** Sh-SNHG14 inhibits cell migration and invasion in CRC.** SW480 and HT-29 cells were transfected with sh-SNHG14. **(A, B)** Scratch test and Transwell chamber assay for the migration and invasion abilities. **(C)** Western blot analysis of the expression levels of migration- and invasion-associated proteins (Cox-2, MMP-2 and MMP-9). Values are mean ± SE, **P<0.01 *vs.* sh-NC, n=3 per group.

As shown in Fig. 3A and 3B, scratch and transwell chamber assays showed that the migration and invasion abilities of SW480 and HT-29 cells were remarkably restrained in cells transfected with sh-SNHG14, when compared to scramble control. Besides, western blot analysis revealed that sh-SNHG14 downregulated the expression of migration and invasion-related proteins (Cox-2, MMP-2 and MMP-9) in SW480 and HT-29 cells (Fig. 3C). All these results illustrated that sh-SNHG14 inhibited migration and invasion of CRC cells.

### SNHG14 sponges and targets on the miR-519b-3p in CRC cells

**Figure 4:**
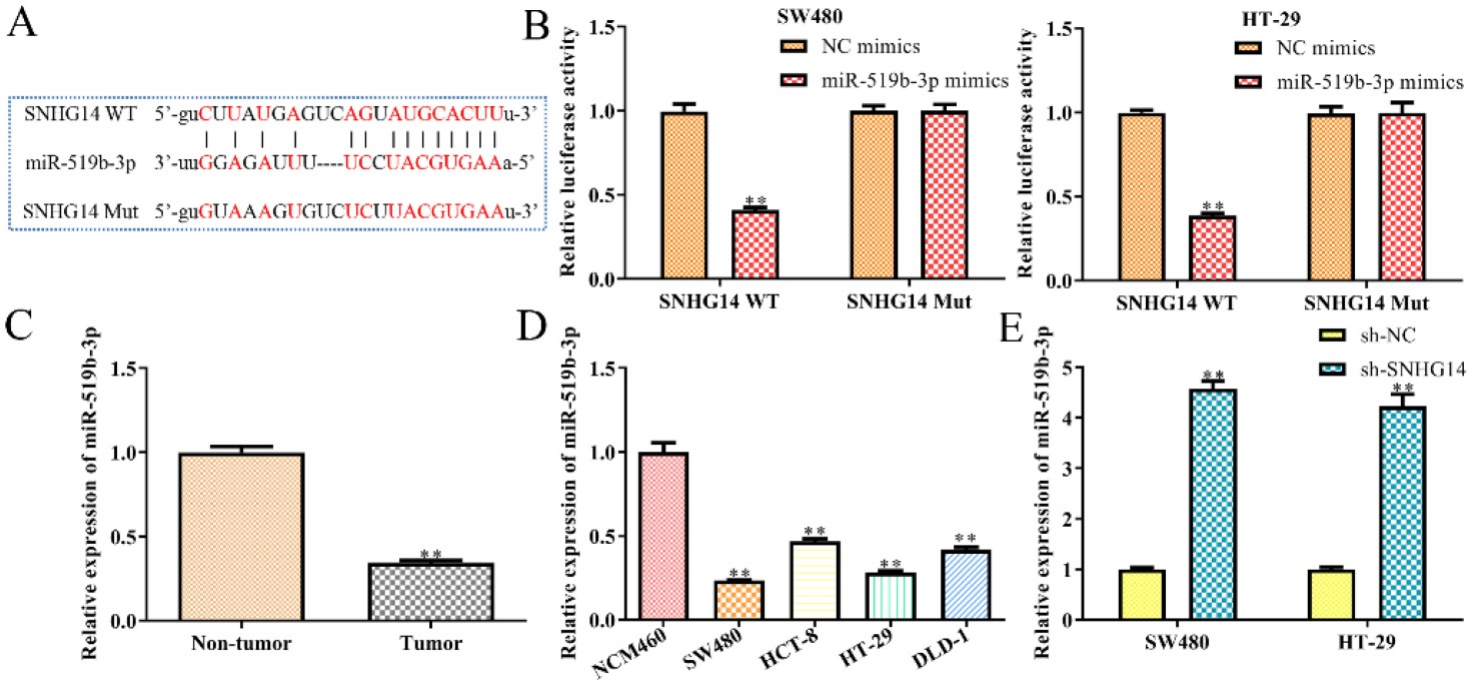
** SNHG14 functions as a sponge for miR-519b-3p in CRC cells. (A)** Bioinformatic analysis of the predicted binding site between SNHG14 and miR-519b-3p. **(B)** Luciferase reporter assay suggested that miR-519b-3p mimics reduced the luciferase activity of SNHG14-WT in SW480 and HT-29 cells, **P<0.01 *vs.* NC mimics. **(C)** qRT-PCR analysis of the expression of miR-519b-3p in tumor tissues and adjacent non-tumor tissues (n= 6 per group), **P<0.01 *vs.* Non-tumor tissue. **(D)** qRT-PCR analysis of the expression of miR-519b-3p in CRC cell lines (SW480, HCT-8, HT-29 and DLD-1) and normal human colon epithelial cells (NCM460), **P<0.01 *vs.* NCM460. **(E)** qRT-PCR analysis of the expression of miR-519b-3p in SW480 and HT-29 cells transfected with sh-SNHG14, **P<0.01 *vs.* sh-NC. Values are mean ± SE, n=3 per group.

Then we studied the molecular mechanism of SNHG14 in CRC. Previous studies have shown that lncRNAs could serve as ceRNAs by sponging miRNAs 13, 14. Hence, we used StarBase analysis to predict the potential miRNA, which interacted with SNHG14, and finally selected miR-519b-3p as a potential target (Fig. 4A), since its dual functions as either oncogene or tumor suppressor in the regulation of the proliferation, migration and apoptosis of tumor cells 15. To evaluate the interactions between miR-519b-3p and SNHG14, luciferase reporter assay was performed and revealed a significant decrease in luciferase activity of mutant SNHG14 in cells co-transfected with SNHG14-WT and miR-519b-3p mimic (Fig. 4B). However, no significant difference was observed in luciferase activity between the miR-519b-3p mimic group and control group when the putative binding sites were mutated (Fig. 4B). Consistently, sh-SNHG14 could increase miR-519b-3p expression in SW480 and HT-29 cells (Fig. 4E). Subsequently, we discovered that miR-519b-3p expression was notably upregulated in non-tumor groups compared to tumor tissues (Fig. 4C). Moreover, RT-PCR indicated that miR-519b-3p expression was remarkably decreased in CRC cells (SW480, HCT-8, HT-29 and DLD-1) compared to NCM460 cells (Fig. 4D). These findings revealed that SNHG14 functions as a sponger for the miR-519b-3p in CRC cells.

### MiR-519b-3p inhibits cell proliferation, migration/invasion and promotes apoptosis in CRC cells

**Figure 5:**
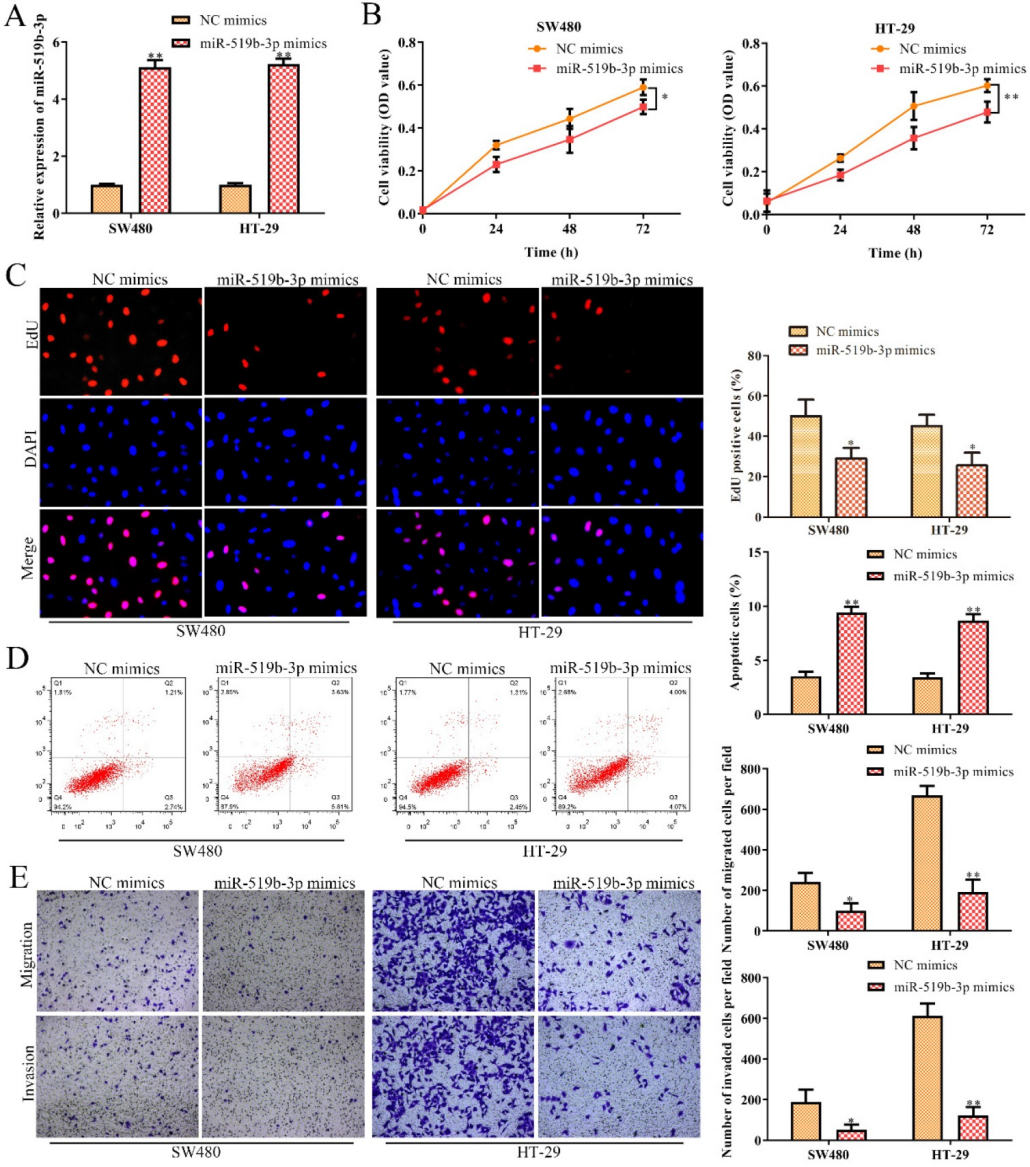
** MiR-519b-3p inhibits cell proliferation, migration, invasion and promotes cell apoptosis in CRC.** SW480 and HT-29 cells were transfected with miR-519b-3p mimics. **(A)** qRT-PCR analysis of the expression of miR-519b-3p, **P<0.01 *vs.* NC mimics. **(B, C)** CCK-8 and EdU assays for the cell proliferation. *P<0.05, **P<0.01 *vs.* NC mimics. **(D)** Flow cytometry analysis of the apoptosis rate. (E) Transwell chamber assay for the migration and invasion abilities, *P<0.05, **P<0.01 *vs.* NC mimics. Values are mean ± SE, n=3 per group.

In order to dissect the biological function of miR-519b-3p in CRC progression, miR-519b-3p mimic and NC mimic were transfected into SW480 and HT-29 cells, respectively. qRT-PCR assay was used to confirm the overexpression efficiency of miR-519b-3p mimic in SW480 and HT-29 cells. We found that miR-519b-3p mimic triggered an obvious upregulation of miR-519b-3p expression in SW480 and HT-29 cells, when compared to NC mimic (Fig. 5A). CCK-8 and EdU assays indicated that miR-519b-3p mimic remarkably decreased the proliferation of SW480 and HT-29 cells (Fig. 5B-C). In addition, flow cytometry assay elucidated that miR-519b-3p overexpression promoted cell apoptosis in SW480 and HT-29 cells (Fig. 5D). Transwell chamber assay further demonstrated that the migration and invasion abilities of cells were obviously repressed in SW480 and HT-29 cells transfected with miR-519b-3p mimic (Fig. 5E). All these results illustrated that miR-519b-3p overexpression inhibited the cell proliferation, migration/invasion, while promoted the cell apoptosis in CRC.

### DDX5 is a downstream target gene of miR-519b-3p

**Figure 6:**
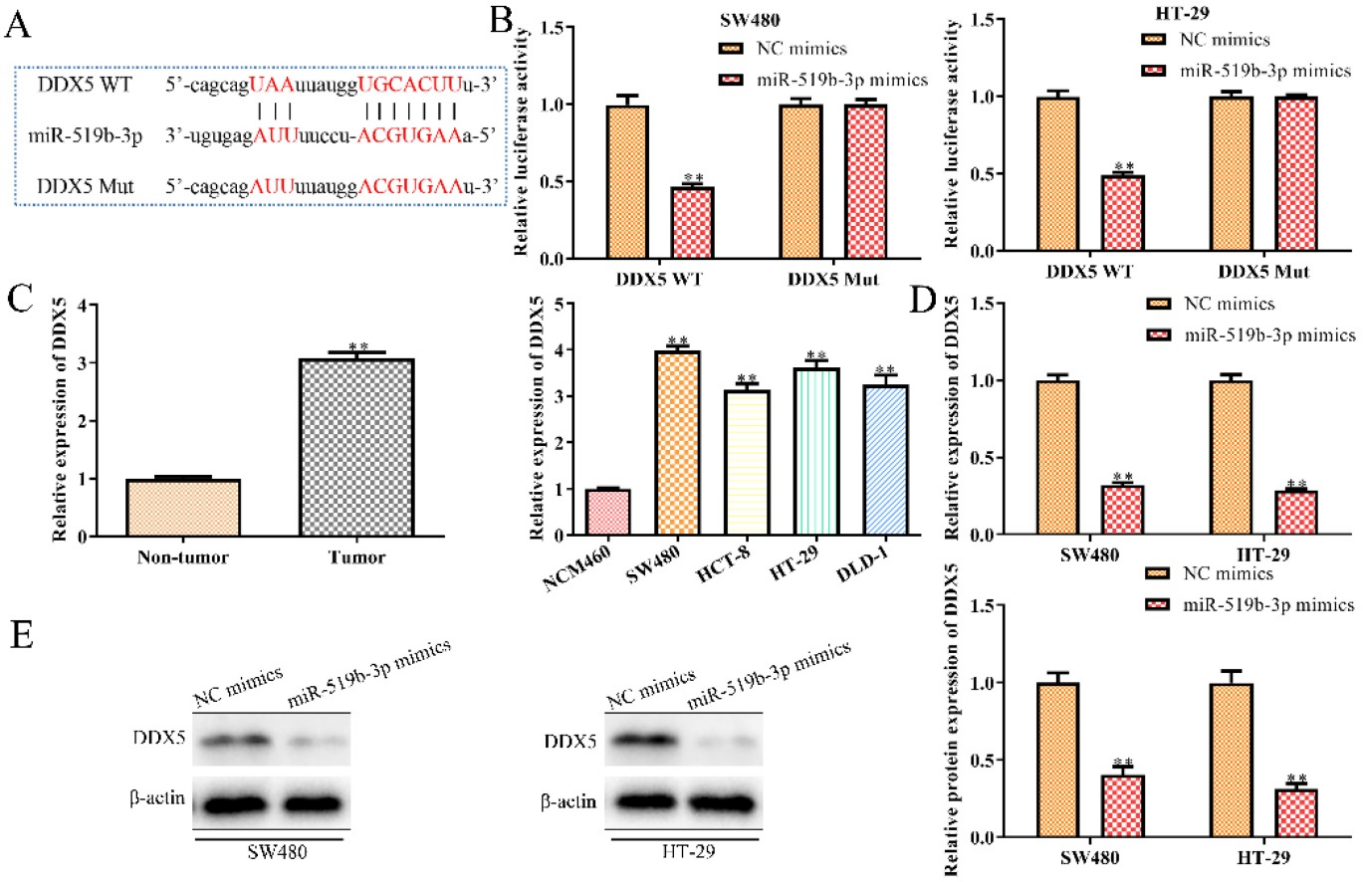
** DDX5 is a downstream target gene of miR-519b-3p. (A)** Bioinformatic analysis of the predicted binding sites of DDX5 and miR-519b-3p. **(B)** Luciferase reporter assay was performed at 48 h after transfection in SW480 and HT-29 cells with luciferase reporter plasmid containing WT or mutant form of DDX5 3′-UTR along with control or miR-519b-3p mimics. **(C)** qRT-PCR analysis of the expression of DDX5 in SW480 and HT-29 cells transfected with miR-519b-3p mimics. **(D)** Western blot analysis of the expression of DDX5 protein. **P<0.01 *vs.* NC mimic, n=3 per group. **(E)** qRT-PCR analysis of the expression of DDX5 in CRC tissues and cell lines. **P<0.01 *vs.* Non-tumor tissue or NCM460 cells, n=3 per group. Values are mean ± SE.

To explore the regulatory mechanism through which miR-519b-3p affected CRC progression, we used TargetScan to predict miR-519b-3p target genes and identified DDX5 as a potential target (Fig. 6A), because of its proto-oncogenic role in human cancers 16-18. To determine the interaction of miR-519b-3p and DDX5, we performed luciferase reporter analysis and found significantly reduction in the luciferase activity of 3'UTR region of DDX5 when the cells were co-transfected with DDX5‑WT and miR-519b-3p mimics. However, there was no notable difference in luciferase activity of DDX5‑Mut between miR-519b-3p mimics group and control group (Fig. 6B). To further study the correlation of DDX5 and miR-519b-3p, we detected that DDX5 expression was decreased by miR-519b-3p mimics in SW480 and HT-29 cells via qRT-PCR and western blot (Fig. 6C-D). In addition, we found that DDX5 expression was significantly upregulated in tumor tissues compared to non-tumor groups, and increased in CRC cells (SW480, HCT-8, HT-29 and DLD-1) compared to NCM460 cells (Fig. 6E). These results demonstrated that DDX5 is a downstream target gene of miR-519b-3p.

### MiR-519b-3p/DDX5 axis mediates the SNHG14 knockdown-induced inhibition of CRC progression

**Figure 7:**
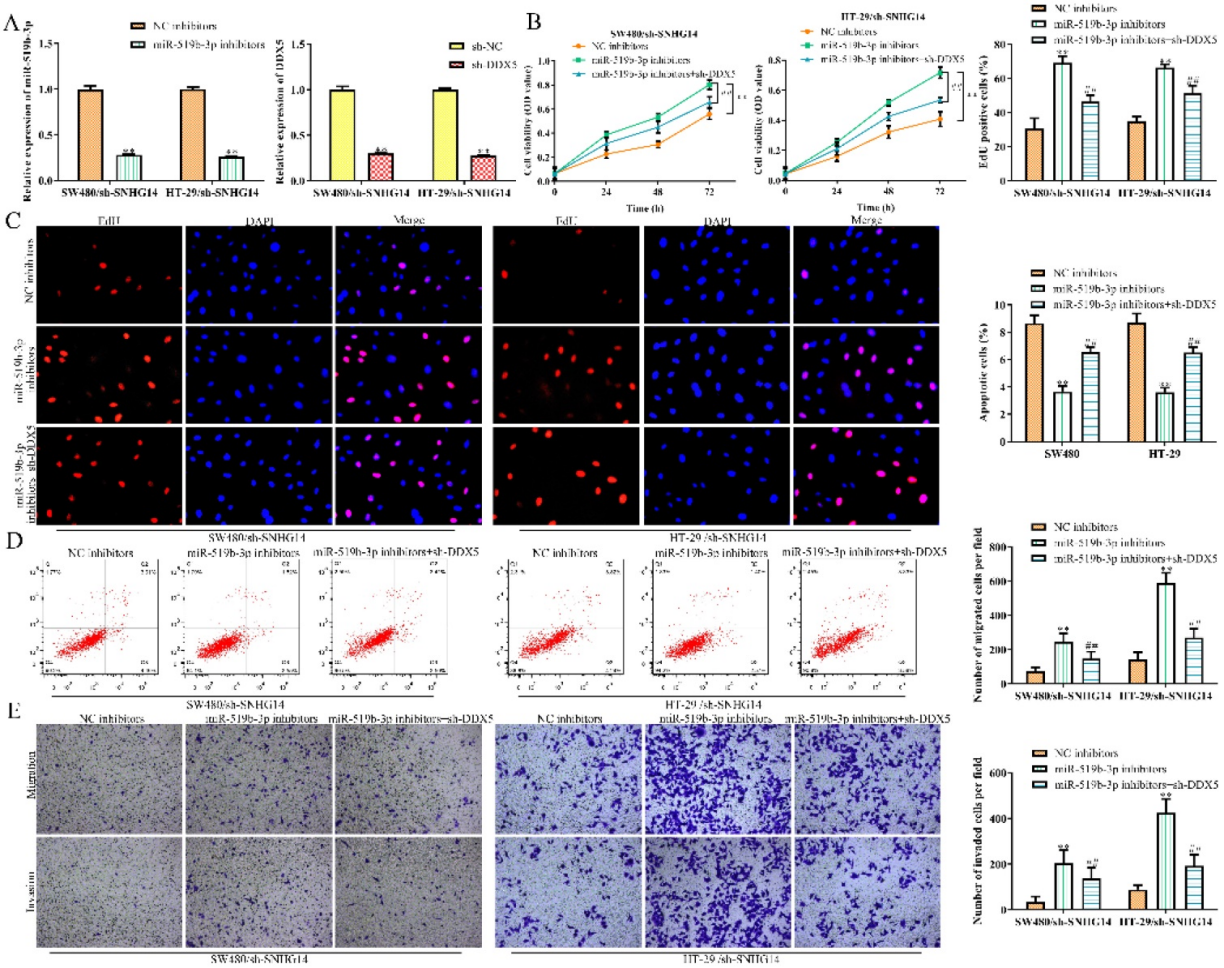
** SNHG14 affects CRC cell proliferation, migration, invasion and apoptosis via regulating miR-519b-3p/DDX5. (A)** qRT-PCR analysis of the expression of miR-519b-3p and DDX5 in SW480 and HT-29 cells transfected with miR-519b-3p inhibitors or sh-DDX5, **P<0.01 *vs.* NC inhibitors, **P<0.01 *vs.* sh-NC. **(B)** CCK-8 assay to detect cell viability of SW480 and HT-29 cells transfected with miR-519b-3p inhibitors or/and sh-DDX5, **P<0.01 *vs.* NC inhibitors, ^##^ *P*<0.01 *vs.* miR-519b-3p inhibitors. **(C)** EdU assay to analyze the cell proliferation of SW480 and HT-29 cells transfected with miR-519b-3p inhibitors or/and sh-DDX5. **(D)** Flow cytometry analysis of the apoptosis rate of SW480 and HT-29 cells transfected with miR-519b-3p inhibitors or/and sh-DDX5, **P<0.01 *vs.* NC inhibitors, ^##^ *P*<0.01 *vs.* miR-519b-3p inhibitors. **(E)** Transwell chamber assay for the migration and invasion abilities of SW480 and HT-29 cells transfected with miR-519b-3p inhibitors or/and sh-DDX5, **P<0.01 *vs.* NC inhibitors, ^##^ *P*<0.01 *vs.* miR-519b-3p inhibitors. Values are mean ± SE, n=3 per group.

To explore whether SNHG14 promoted the CRC progression through regulating miR-519b-3p/DDX5 pathway, we performed rescue experiments using miR-519b-3p inhibitors and sh-DDX5. qRT-PCR assay was used to confirm the inhibition efficiency of miR-519b-3p inhibitors and sh-DDX5 in SW480 and HT-29 cells transfected with sh-SNHG14. We found that miR-519b-3p and DDX5 expression was downregulated treated with miR-519b-3p inhibitors and sh-DDX5 in SW480/sh-SNHG14 and HT-29/sh-SNHG14 cells (Fig. 7A). CCK-8 and EdU assays demonstrated that sh-DDX5 could rescue miR-519b-3p inhibitors-induced activation of the proliferation of SW480/sh-SNHG14 and HT-29/sh-SNHG14 cells (Fig. 7B-C). In addition, flow cytometry assay showed the apoptosis of the SW480/sh-SNHG14 and HT-29/sh-SNHG14 cells was decreased when these cells were transfected with miR-519b-3p inhibitors, whereas sh-DDX5 reversed the reduction of apoptosis caused by miR-519b-3p inhibitors (Fig. 7D). Transwell chamber assay illustrated that sh-DDX5 reversed miR-519b-3p inhibitors-induced activation of the migration of SW480/sh-SNHG14 and HT-29/sh-SNHG14 cells (Fig. 7E). Based on the results, we concluded that SNHG14 promotes the CRC progression by miR-519b-3p/DDX5 axis.

### Sh-SNHG14 inhibits the CRC progression *in vivo*

**Figure 8:**
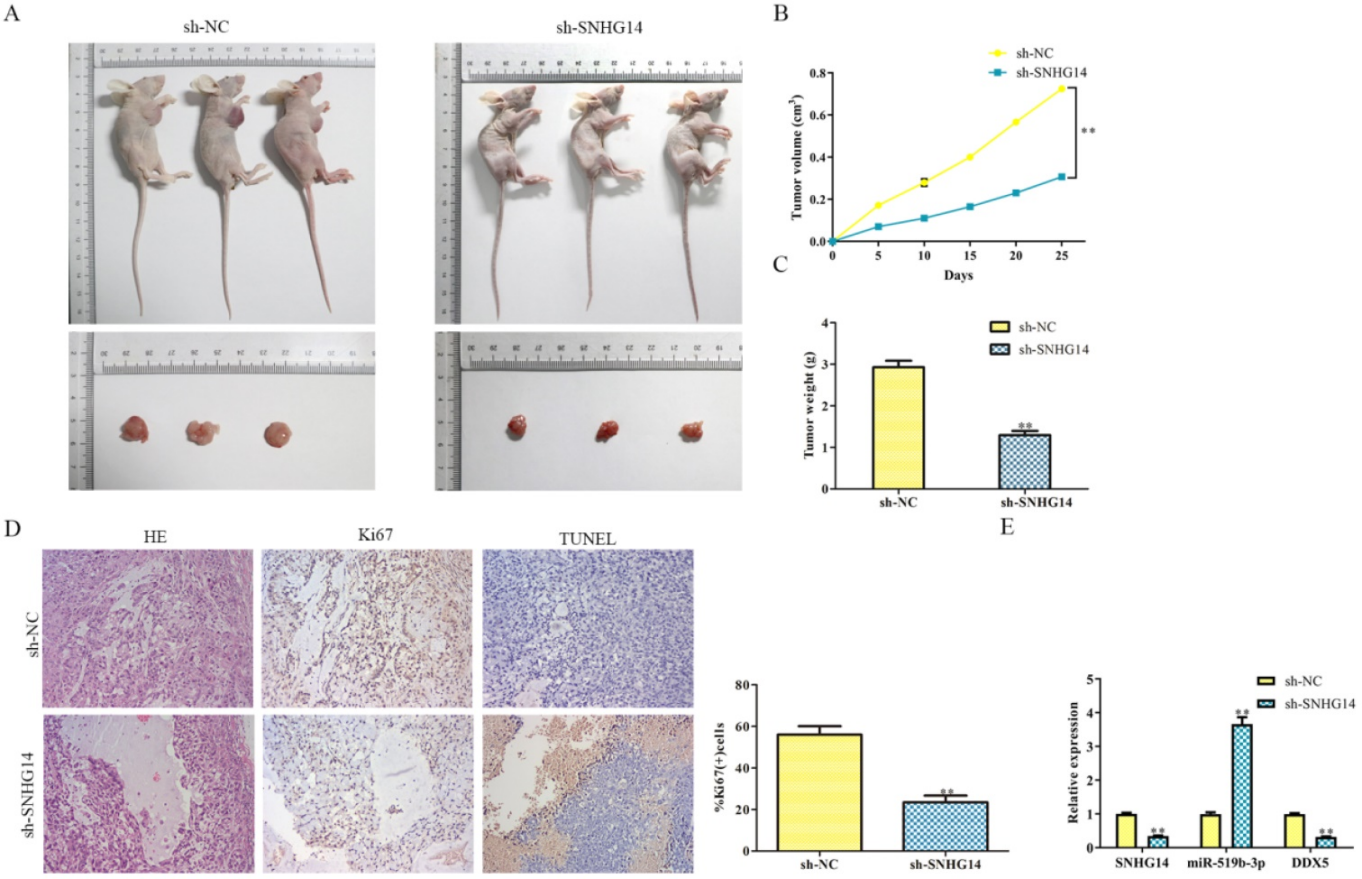
** Sh-SNHG14 inhibits the CRC progression* in vivo*. (A)** Tumor growth of experimental NOD-SCID mice models after injected with HT-29 cells transfected with sh-SNHG14. **(B, C)** Tumor volume and weights of the mice at the end of 25 days. **(D)** Representative Ki67 and TUNEL level in subcutaneous tumors of the mouse model. (E) Obtained tumor samples were dissociated for qRT-PCR analysis and the results exerted the SNHG14, miR-519b-3p and DDX5 expression. Values are mean ± SE, **P<0.01* vs.* sh-NC, n=6 per group.

Given the suppressive role of sh-SNHG14 *in vitro*, we supposed to find out its impact on tumor growth *in vivo*. Human CRC cell lines were transfected by sh-SNHG14/sh-NC, the transfected HT-29 cells were injected subcutaneously into NOD-SCID mice to observe its tumorigenicity. As shown in Fig. 8A and 8B, we found that tumors from sh-SNHG14 transfected groups were obviously larger than sh-NC transfected groups. Throughout the growth of tumor, tumor lumps from the sh-SNHG14 transfected groups developed slower than those from sh-NC transfected groups (Fig. 8C). Moreover, HE staining results showed that the tumor cells are loosely from the sh-SNHG14 transfected groups compared with sh-NC transfected groups. TUNEL staining results suggested that sh-SNHG14 significantly promoted CRC cell apoptosis, and the immunohistochemistry results also showed that Ki67 protein levels in the tumor tissues generated from sh-SNHG14 transfected cells were strongly reduced (Fig. 8D). qRT-PCR results showed that sh-SNHG14 induced the decreased the expression of SNHG14, increased the expression of miR-519b-3p and decreased the expression of DDX5 (Fig. 8E). Collectively, we concluded that sh-SNHG14 could inhibit CRC tumor growth *in vivo*.

## Discussion

LncRNAs are crucial regulators involved in the gene expression and cancer development 9, 19, 20. Therefore, the investigation in demonstrating the role of lncRNA could provide new insights into the identification of potential therapeutic targets in clinical applications. It is reported that lncRNA SNHG14 involved in procession of many cancers, such as pancreatic ductal adenocarcinoma, cervical cancer, renal cell carcinoma and non-small cell lung cancer, and so on 21-23. For example, Zhang Z et al. found that SNHG14 exerts oncogenic functions in non-small cell lung cancer by acting as an miR-340 sponge 21. However, the function and mechanism of SNHG14 in CRC remain elusive. Currently, it was reported that SNHG14 stimulated cell autophagy to facilitate cisplatin resistance of CRC by regulating miR-186/ATG14 axis 24. In addition, SNHG14 had been reported to facilitate CRC metastasis through EZH2/EPHA7 25. In the current investigations, we observed that SNHG14 was highly expressed in CRC tissues as well as in the CRC cell lines, especially in SW480 and HT-29 cells. Moreover, functional assays showed that sh-SNHG14 markedly repressed cell proliferation, cell migration/invasion, and promoted cell apoptosis in CRC cell lines. All these data demonstrated that SNHG14 promoted the progression of CRC.

Accumulating evidence has shown that miRNAs act as critical regulators of cancer pathogenesis and progression 26, and lncRNAs could function as competing endogenous RNAs (ceRNAs) by sponging miRNAs to regulate the expression of specific genes targeted by miRNA, and regulate the progression of cancers. For instance, lncRNA ATB promotes the invasion-metastasis cascade in hepatocellular carcinoma 27. LncRNA Hotair mediates tumorigenesis and metastasis in hepatocellular carcinoma through acting as an endogenous sponge for miRNA-218 and activating P14 and P16 signaling 28. On the other hand, miR-519b-3p, a novel cancer-associated miRNA, is frequently dysregulated in a variety of diseases and plays a critical role in cellular physiology. Previous researches indicated that miR-519b-3p promotes responsiveness to preoperative chemoradiotherapy in rectal cancer patients by targeting ARID4B 29. MiR-519b-3p inhibits the proliferation and invasion of colorectal cancer by regulating the uMtCK/Wnt signaling pathway 30. All these studies illustrated that scientists extensively focused on the downstream mechanism of miR-519b-3p in CRC, but the upstream regulatory mechanism of miR-519b-3P is almost unknown until now. In our study, miR-519b-3p was experimentally confirmed as a target of SNHG14, and miR-519b-3p expression was down‐regulated in CRC tissues and cell lines, which correlated negatively with the expression of SNHG14. Additionally, miR-519b-3p OE inhibits cell proliferation, migration, and invasion, as well as increased cell apoptosis rates in CRC. Similar to previous study that SNHG14 could accelerate cell proliferation, migration, invasion and suppress apoptosis in CRC cells by targeting miR-944 31, our data suggested that SNHG14 regulated CRC progression by sponging miR-519b-3p.

In order to further explore the downstream mechanism of miR-519b-3p, we then performed bioinformatics analysis, and DDX5 was predicted as a possible target of miR-519b-3p. DDX5 exerts crucial roles in several molecular processes, including transcription, pre-mRNA processing, and miRNA processing 32. It is reported that DDX5 plays tumor-promoter role in various cancers 33. For instance, Du et al. 34 found that DDX5 was up-regulated in gastric cancer tissues, promoting gastric cancer progression through mTOR pathway. Ma et al. 35 observed significantly overexpressed DDX5 in human esophageal cancer cell lines while silencing of DDX5 inhibit the tumorigenesis in esophageal cancer via Wnt/β-catenin pathway. Another research by Wang et al. 36 illustrated that DDX5 was increased in tumor tissues from lung cancer patients, playing tumor-promotive role in lung cancer. In the realm of CRC, from a cohort analysis downloaded from Starbase (http://starbase.sysu.edu.cn/starbase2/index.php) containing 471 cancer samples and 41 normal samples, DDX5 was significantly up-regulated in CRC samples (Supplementary file). Besides, Zhang et al. 37 and Dai et al. 38 illustrated that DDX5 was highly expressed in CRC, exerting its oncogenetic role in CRC development. Consistent with previous investigation, in our work, DDX5 was experimentally confirmed as a target of miR-519b-3p, and we found DDX5 expression was significantly upregulated in CRC and negatively correlated with miR-519b-3p. Moreover, sh-DDX5 partially rescued SNHG14/miR-519b-3p knockdown-mediated effects on cell proliferation, migration/invasion, and apoptosis in CRC cell lines. Finally, *in vivo* experiments further indicated that down-regulated SNHG14 greatly inhibited CRC tumor growth.

However, there were still some limitations in our study. First, from the cohort results downloaded from Starbase (http://starbase.sysu.edu.cn/starbase2/index.php), we found that SNHG14 was decreased in CRC tumor samples compared with normal samples (Supplementary file), which was inconsistent with our results. In the previous studies, we also observed contradictory results concerning the expression of SNHG14 in CRC tissues. Consistent with our results, Di et al. 25, Ye et al. 39, Han et al. 24 and Pei et al. 31 verified that SNHG14 was up-regulated in CRC tumor tissues or/and cell lines, functioning as a tumor-promoter in CRC. However, Zhang et al. 40 suggested that SNHG14 was low-expressed in CRC tumor tissues and correlated with poor prognosis. Throughout various studies in cancer realm, we could conclude that SNHG14 functioned as an oncogenetic role during tumorigenesis, including endometrial cancer 41, diffuse large B cell lymphoma 42, ovarian cancer 43,44, pancreatic ductal adenocarcinoma 45, cervical cancer 46,47, osteosarcoma 48, hepatocellular carcinoma 49-51, lung cancer 52,53, retinoblastoma 54 and bladder cancer 55. In our study, we only enrolled 30 CRC patients and all the cases were collected from the Second Affiliated Hospital of Nanjing Medical University (Nanjing, China), thereby the results may bias. Hence, a larger number of CRC patients need to be collected in order to improve our results. Moreover, the clinical significance of SNHG14, miR-519b-3p and DDX5 in CRC diagnosis and prognosis should be further investigated in the future.

Taken together, these findings demonstrated that SNHG14 promotes cell proliferation and invasion in CRC through modulating miR-519b-3p/DDX5 axis. Our study facilitates the current understanding of SNHG14 function in CRC and provides a novel therapeutic target for CRC.

## Figures and Tables

**Table 1:** The detailed clinic parameters of enrolled patients

Clinical parameters	SNHG14		P value
	Low expression (n=14)	High expression (n=16)	
**Age**			0.165
> 60	8	9	
≤ 60	6	7	
**Gender**			0.352
Male	8	8	
Female	6	8	
**Tumor Size**			0.026*
> 2 cm	4	7	
≤ 2 cm	10	9	
**Tumor stage**			0.039*
I~II	9	4	
III~IV	5	12	
**Lymph nodes metastasis**			0.068
No	5	6	
Yes	9	10	
**Distant metastasis**			0.028*
No	10	5	
Yes	4	11	
